# Editor's note

**DOI:** 10.1098/rstb.2023.0260

**Published:** 2023-10-23

**Authors:** Philosophical Transactions B editorial team

This is the second of a two-part issue: ‘Causes of obesity: theories, conjectures and evidence’. Part I can be found at https://royalsocietypublishing.org/toc/rstb/2023/378/1885. The Preface to Part I contains an introduction to both issues [1].
